# Pityriasis Lichenoides Chronica of Esophagus: A Rare Case Report

**DOI:** 10.7759/cureus.32290

**Published:** 2022-12-07

**Authors:** Muhammad Tahir, Osama Elkadi, Shou S Liu, Sandhyarani Dasaraju

**Affiliations:** 1 Pathology and Laboratory Medicine, University of South Alabama Health Hospital, Mobile, USA; 2 Pathology and Laboratory Medicine, Larkin Community Hospital, South Miami, USA; 3 Pathology and Laboratory Medicine, University of Minnesota, Minneapolis, USA

**Keywords:** lichenoid-like lesion, lichenoid disorders, esophageal stricture, oral lichen planus, pityriasis lichenoides chronica

## Abstract

Pityriasis lichenoides chronica (PLC) is an uncommon inflammatory skin condition of unknown cause that ranges from a mild chronic form to a more severe acute eruption. Both forms usually involve the skin of the trunk and proximal extremities, and visceral involvement is not a well-described phenomenon.

Here, we report a case of PLC that presented with esophageal involvement that occurred after a period of discontinuation of PLC treatment. The histological pattern of involvement is in the form of lymphocytic esophagitis, a non-specific pattern with a broad differential diagnosis. Awareness of the potential involvement of the esophagus and attention to certain endoscopic and morphological details may better help classify esophagitis biopsies and the diagnosis of this rare non-neoplastic chronic inflammatory disease. To our knowledge, this is the first-ever case of PLC with esophageal involvement, and nothing has been reported in the English literature earlier.

## Introduction

Pityriasis lichenoides, an uncommon interface dermatitis, is an inflammatory skin condition of unknown cause that ranges from mild chronic form to a more severe acute eruption. The mild form, pityriasis lichenoid chronica (PLC) or guttate parapsoriasis is characterized by the gradual development of non-symptomatic, small brown-red scaling papules that spontaneously flatten and regress over weeks or months [1]. The acute form is characterized by the sudden eruption of small-scaling papules that develops into blisters and crusted red-brown spots. This acute condition is referred to as pityriasis lichenoides et varioliformis acuta (PLEVA) and its most severe form is known as febrile ulceronecrotic Mucha-Habermann disease. Histologically, PLEVA presents as a brisk perivascular lymphocytic infiltrate with interface change and wedge-shaped reticular dermis extension, whereas PLC demonstrates blunted features of PLEVA with focal apoptotic keratinocytes [2]. Both forms usually involve the skin of the trunk and proximal extremities. Although certain interface dermatitis, such as lichen planus, can simultaneously present with esophageal manifestation, visceral mucosal involvement in PLC is not a well-described phenomenon, and no esophageal involvement has been previously reported in the literature [2-4].

## Case presentation

The patient is a 69-year-old female with a significant history of cutaneous PLC, hyperlipidemia, and endometriosis. Her past surgical history is significant for appendectomy, hysterectomy, cholecystectomy, and colonoscopy. Her PLC has been well-controlled for the past one and half years by immunosuppressive therapy, Methotrexate sodium 2.5 mg/6 weekly. Apart from immunosuppressive therapy, she is also on clotrimazole, folic acid, prednisolone acetate, vitamin D, and tropical neomycin-polymyxin-HC 3.5-10,000-10 mg-unit-mg/ml/daily.

Recently, the patient presented with odynophagia, dysphagia, and cough. Further questioning revealed that the patient has difficulty continuing her PLC medication because of dysphagia. She also reported very infrequent symptoms of pyrosis one time/month treated by over-the-counter antacids. The patient also reported multiple episodes of oral thrush treated with Diflucan and oral mouthwash. Due to her bothersome oral symptoms, an upper endoscopy examination was performed. Endoscopy showed diffuse edema and the mid and distal esophagus was dry, erythematous, and narrowed due to the presence of strictures. Random, multiple, cold-forceps upper esophageal biopsies were obtained.

**Figure 1:**
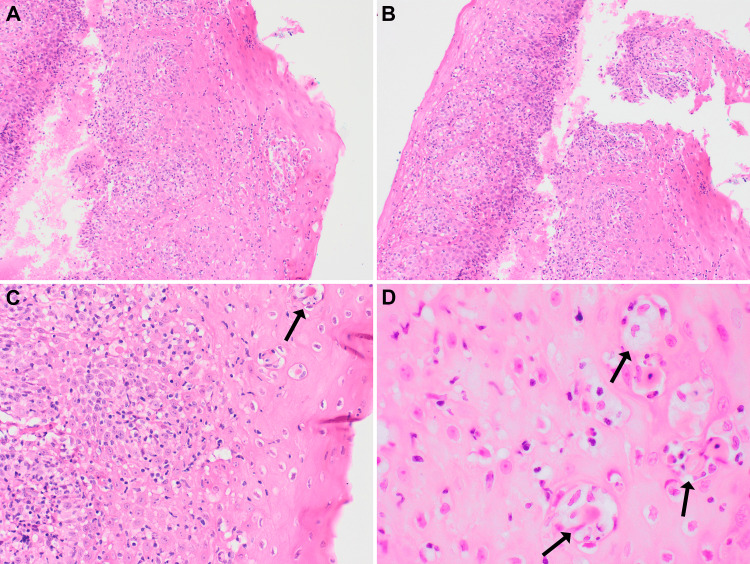
A and B: Low-power view (2x) showing esophageal squamous mucosa with band-like basal inflammatory infiltrate. C: Medium-power view (10x) showing acanthosis, basal cell hyperplasia, intraepithelial lymphocytosis, and apoptotic keratinocytes (black arrow). D: High-power view (40x) showing focal prominent apoptotic keratinocytes (black arrows)

**Figure 2:**
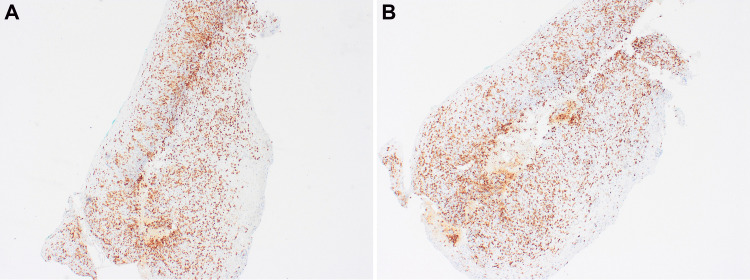
(2x) low power view showing CD3 (A) and CD8 (B) positive cytotoxic T-lymphocytes

Grossly, the biopsy specimen consisted of a few, tiny, tan-pink esophageal mucosal fragments. Microscopic evaluation revealed multiple fragments of esophageal squamous mucosa showing a band-like inflammatory infiltrate (Figure 1A), with diffuse lymphocytes exocytosis (intraepithelial lymphocytosis), acanthosis, basal cell hyperplasia with prominent apoptotic keratinocytes identified (Figures 1B-1D). The inflammatory infiltrate was composed predominantly of CD3+ T-lymphocytes with scattered plasma cells and macrophages. There was no significant acute inflammation and no increase in intraepithelial eosinophils. Immunohistochemistry stains for CD3, CD8, and CD20 showed the majority of inflammatory cells consisting of CD3-positive T-lymphocytes with a predominance of CD8-positive cytotoxic T-cells (Figures 2A-2B). No viral or fungal organisms were demonstrated on periodic-acid-Schiff (PAS), cytomegalovirus (CMV), and herpes simplex virus type 1 (HSV-1) stains.

## Discussion

The esophagus is frequently involved in several primary dermatologic conditions including lichen planus, lichen sclerosis, acanthosis nigricans, and bullous skin diseases. Other conditions that may involve the skin and the esophagus include infections, connective tissue diseases, drug-induced injury, Crohn’s disease, and graft-versus-host disease. Esophageal involvement may coincide with or occur after dermatological manifestations. Rarely, esophageal involvement occurs in the absence of dermatologic disease or may even precede it [2-3].

Clinical manifestations of esophageal involvement by skin diseases include dysphagia, odynophagia, and stricture formation sometimes leading to weight loss and findings concerning for malignancy. Malignant transformation has been rarely reported in association with esophageal lichen planus [5]. It is, however, speculated that most of the cases of esophageal involvement in dermatologic diseases are clinically silent, and the condition is significantly underreported and underdiagnosed. Rarely, hematemesis and death have been reported secondary to lichen planus and certain blistering diseases [6].

Endoscopic examination of the esophagus involved in skin conditions mostly shows nonspecific findings, including stricture, white papules, pinpoint erosions, esophageal pseudo-membranes, and inflamed mucosa [3]. These findings may simulate reflux esophagitis, eosinophilic esophagitis, and fungal or viral esophagitis. Therefore, a biopsy is necessary to distinguish this cutaneous-esophageal condition from other primary esophageal disorders. The histologic pattern of involvement is often in the form of “lymphocytic esophagitis,” referring to an abundance of intraepithelial lymphocytes and associated marked spongiosis. The term “lichenoid esophagitis” was primarily proposed by Salaria et al. for a pattern of inflammation in the esophagus, which is similar to lichenoid interface dermatitis [2]. It is characterized by a T-lymphocyte predominant subepithelial band-like inflammatory infiltrate, increased intraepithelial lymphocytosis, acanthosis, and single necrotic squamous cells (Civatte bodies). This pattern is not specific, however, and it has been described in association with drugs, viral infections, esophageal Crohn’s disease, and pill-induced esophagitis in addition to lichen planus as well as in our case of PLC.

Pityriasis lichenoides is a term used to describe the unique group of acquired inflammatory skin disorders that includes pityriasis lichenoides chronica (PLC), pityriasis lichenoides et varioliformis acuta (PLEVA), and febrile ulceronecrotic Mucha-Habermann disease (FUMHD) variant of PLEVA. PLC is clinically characterized by the development of multiple scaly, asymptomatic red-brown, erythematous, small-sized papulosquamous lesions appearing in crops commonly involving the buttocks, trunk, and upper extremities. The condition usually has a relapsing and remitting course that persists for months or years [7].

The epidemiology of PLC is inconclusive due to limited data in the published literature and the different clinical subtypes sharing similar presentations. PLC typically affects children and young adults but can develop at any age, including in older individuals. [8]. At present, the pathogenesis of PLC is unknown and poorly understood. However, it is hypothesized that some parasitic infections (Toxoplasma gondii), viral (Epstein-Barr virus, parvovirus B16, HIV), and bacterial infections (staphylococci, Group A streptococci) might be the trigger for the development of PLC [9-11]. The characteristic histopathological features of cutaneous PLC include acanthosis, parakeratosis, mild spongiosis, sparse necrotic keratinocytes, hyperplasia of the basal cell layer, lymphocytes exocytosis, and lichenoid band-like perivascular lymphohistiocytic infiltrate in the superficial dermis and epidermis [12].

The diagnosis of esophageal PLC requires a biopsy and relies on clinical history, endoscopic findings, and histopathological features. The morphology shares significant overlapping features with other forms of lymphocytic (lichenoid) esophagitis.

A few skin conditions that mimic PLC, their clinical presentation, and potential esophageal involvement, in addition to therapeutic options, are listed in Table 1 [13].

**Table 1:** Differentials diagnosis of PLC PLC: pityriasis lichenoides chronica; SN: serial number

SN	Name	Clinical Features	Sign/Symptoms	Most common sites/ esophageal involvement	Histological findings	Treatment
1	Pityriasis lichenoides et varioliformis acuta (PLEVA)	Acute eruption of multiple erythematous macules, inflammatory papules, and vesicles. Some degree of hemorrhagic crusting is common.	Fever and arthralgia, pruritus, burning sensation, and may be asymptomatic.	Trunk, proximal extremities, and skin at the flexure areas are the most common sites. However, any cutaneous surface can be affected. Mucosal involvement is typically absent. To our knowledge, no cases of PLEVA involving the esophagus have been reported in the literature.	Similar histologic features to PLC, with a more brisk, inflammatory infiltrate and fewer apoptotic keratinocytes.	Antibiotics, phototherapy, and topical corticosteroids.
2	Pityriasis rosea (PR)	PR is a disease of older children and young adults and is more common in females than in men. The eruption begins with the “mother” or “Herald” patch, a single, oval to round, pink to salmon colored, 2 to 5 cm in diameter skin lesion with sharp edges, which appears first on the chest, neck, and back.	A prodrome of headache, malaise, and pharyngitis may occur in a small number of cases, but except for itching, the condition is usually asymptomatic.	Scalp, face, and distal extremities while sparing the trunk, or in some instances may be concentrated in the pubic, inguinal, and axillary regions. To our knowledge, no cases of PR involving the esophagus have been reported in the literature.	Picture of subacute spongiotic dermatitis with perivascular lymphocytic infiltrate, acanthosis, parakeratosis, and rare dyskeratotic keratinocytes.	Topical corticosteroids, antibiotics, antivirals, and phototherapy.
3	Guttate psoriasis	The term "guttate" refers to the discrete, drop-like appearance of skin lesions. Guttate psoriasis is characterized by sudden de novo eruption of 2 to 15 mm in diameter, numerous erythematous papules, and plaques	Pruritus, dry flaking scales, and post-inflammatory hyperpigmentation.	The classic sites are the trunk and proximal extremities however, the lesion may occur in other sites such as the scalp, hands, nails, and feet. No cases of esophageal psoriasis had been identified. Instead, a case of esophageal squamous cell carcinoma in a patient with a past medical history of psoriasis was reported [14].	Acanthosis, elongation of rete ridges, papillary dermal edema, Munro micro-abscess, and superficial perivascular infiltration of lymphocytes, neutrophils, and macrophages.	Phototherapy, topical corticosteroids, and vitamin D analogs.
4	Lichen planus (PL)	LP is a rare disease of unknown etiology that mostly targets middle-aged adults. The hypothesis of its association with hepatitis C is controversial and debatable.	The classic four “P’s" of LP: 1-Pruritic, 2-Purple, 3-Polygonal, 4-Papules or plaques	LP may affect the skin and mucus membranes, especially the oral mucosa, nails, scalp, and genitalia. The ankles and volar surface of the wrists are common sites of cutaneous LP. LP may involve the esophagus, producing strictures, scarring, or ulceration.	Cutaneous LP shows acanthosis, intraepithelial lymphocytosis with prominent, band-like lymphocyte infiltrate in the lamina propria, dyskeratotic keratinocytes (Civatte bodies), and junctional split at the epithelium and lamina propria junction. Esophageal LP has similar findings as cutaneous LP [15].	Topical, intra-lesional corticosteroids, severe cases, intravenous steroids, phototherapy, and oral retinoids.
5	Syphilis	Syphilis is caused by the bacteria “Treponema pallidum” and most cases are sexually transmitted. Infection is subdivided into early and late infections. Early: Primary syphilis: Benign painless skin lesions at the site of inoculation (Chancre). Secondary syphilis: Rash, severe cutaneous ulcerations called lues maligna. Late: Tertiary syphilis: Gummas formation in HIV individuals, neurosyphilis, general paresis, and tabes dorsalis.	Primary syphilis: Painless skin lesion (chancre). Secondary syphilis: Rash is classically a diffuse, symmetric maculopapular rash involving the entire trunk and extremities, including the palms and soles with cutaneous ulcerations called lues maligna. Tertiary syphilis: ●Cardiovascular syphilis; aortitis; ●Gummatous syphilis; granulomatous, nodular lesions usually skin and bones; ●CNS involvement; general paresis and tabes dorsalis.	Primary syphilis: Localized skin lesions at the inoculation site. Secondary syphilis: Generalized skin rash; Tertiary syphilis: Can involve any bodily systems including cardiovascular and CNS systems. Esophageal involvement is described in secondary and tertiary syphilis.	Any stage of syphilis can affect any part of the body including the esophagus. Esophageal syphilis can present with gummas formation with necrotizing granulomas [16], or rarely shows a picture of lichenoid esophagitis [17].	Antibiotics
6	Lymphomatoid papulosis (LyP)	LyP is of unknown etiology disorder and is part of the group of cutaneous CD30+ lymphoproliferative disorders that has an excellent prognosis. It affects all ethnic groups and has a bimodal age distribution with male predominance in the pediatric population and female predominance in young adults. Patients with LyP have an increased risk of hematologic malignancies throughout their life. The two most common neoplasms include mycosis fungoides (MF) and cutaneous or systemic anaplastic large cell lymphoma (ALCL).	The disease course is divided into different development phases. The early phase lesions are commonly 2 cm, erythematous papules, or nodules that may evolve and become larger and develop central necrosis, hemorrhage, and crusting, and usually regress spontaneously or completely disappear in 3 to 8 weeks. The manifestation of the LyP is highly variable, the eruptions may recur and regress for months, years, or even decades. In complicated cases, patients might exhibit general systemic symptoms, including fever, weight loss, and sweating.	LyP is more common in the trunk and extremities; however, any body part can be affected from head to toe. Oral and mucosal involvement is rare and to our knowledge, no cases of LyP involving the esophagus have been reported in the literature.	Histologically, cutaneous LyP shows acanthosis, intraepithelial lymphocytosis with a prominent band-like lymphocytic infiltrate in the lamina propria, dyskeratotic keratinocytes (Civatte bodies), and junctional split at the epithelium and lamina propria junction.	Immunosuppression such as methotrexate, phototherapy, and anti-CD30- monoclonal antibody.

PLC is a benign disease that is nonscarring, often asymptomatic, and self-limiting in nature. There is no clear consensus and guidelines for the PLC treatment because of a paucity of high-quality efficacy data and the benign course of the disease. Multiple prophylactic treatment modalities are available for including first-line therapy with topical corticosteroids, oral antibiotics, and phototherapy. More aggressive treatment with immunomodulatory agents like methotrexate is reserved for severe refractory cases [18]. In our case, the patient is in remission and actively being treated with topical and oral immunosuppressive therapy as discussed in the case presentation.

## Conclusions

PLC is a rare disorder that imposes diagnostic and therapeutic challenges on clinicians. When this rare entity is clinically suspected, a biopsy is recommended to confirm the diagnosis by histology. PLC does not have a specific treatment, but this condition responds well to topical corticosteroids, immunosuppressants, and phototherapy. Antibiotics can also be used in case of secondary bacterial infections. The purpose of this case report is to provide awareness of esophageal involvement by PLC and to bring attention to certain endoscopic and morphological details of this entity that might better help classify esophagitis causes.
